# Commercial motorcyclists and road safety measures compliance. A case study of Dodoma city, central Tanzania

**DOI:** 10.1016/j.heliyon.2022.e10297

**Published:** 2022-08-19

**Authors:** Jackson Silvano Nzuchi, Selestin Joseph Ngoma, Eugene Benjamin Meshi

**Affiliations:** a Department of Public Health, School of Nursing and Public Health, University of Dodoma, P.O. BOX 259 Dodoma, United Republic of Tanzania; b Department of Criminal Investigation, Ministry of Home Affairs, P.O. BOX 70 Kigoma, United Republic of Tanzania

**Keywords:** Commercial motorcyclists, Road safety measures, Compliance

## Abstract

**Objective:**

Road traffic accidents have been reported to contribute a greater proportion of deaths. Motorcyclists are among the high-risk group within road users to succumb to road traffic accidents. Road traffic accidents reflect the co-occurrence of multiple causes that involve road infrastructure, vehicle, and people. Understanding the level of compliance with road safety measures and their associated factors among commercial motorcyclists is important approach in the steps towards road traffic accident prevention.

**Study design:**

A cross-sectional study of commercial motorcyclists from two districts in Dodoma region was conducted in 2020.

**Methods:**

Multistage sampling method was used to recruit motorcyclists from registered parking stations. At first stage, purposive sampling was used to select the two districts, while in the second stage simple random sampling by lottery method was employed in the selection of wards and parking stations. Descriptive and Chi-square test analyses determined the compliance level. Bivariate and multivariate logistic regression analyses were performed to determine the associated factors.

**Results:**

A total of 378 male commercial motorcyclists participated in the study. Majority (87.8%) were within the age range of 18–29 years. A significant proportion of participants (61.9%) had adequate knowledge and 59.5% showed positive attitude towards road safety measures. Only 39.7% reported to have good level of compliance with road safety measures. Married motorcyclists and those with good road safety practice regarding road safety measures were more likely to comply with road safety measures than their counterpart.

**Conclusion:**

Good road safety practices and marital status were predictors of good compliance with road safety measures. Coordinated approaches, including continuing provision of road safety education and enforcement of road safety measures are needed to control the way motorcyclist behave on the road.

## 1 Introduction

Over 60% of African people will live in urban areas by 2050. Africa's rapid urbanization challenges are, fast-growing urban transport and mobility needs of its citizen, among others [1]. Road safety is among key issues related to the impact of high motorization rates and urban transport [2]. Road safety measures are in place to improve road safety. They may be directed in any element of road system: pattern of land use, traffic control devices, motor vehicles, police enforcement and road users and their behavior [3]. Knowledge and attitude of the road users on the adherence to these measures help to combat road traffic accidents and injuries. Road traffic accidents and injuries are the major public health challenges [4, 5, 6, 7]. Reports from the World Health Organization (WHO) indicate an increasing trend of recorded death and injuries yearly. About 1.25 million, 1.2 million and 1.35 million people died from road traffic accidents (RTAs) in 2013, 2015 and 2018 respectively [8, 9, 10]. Moreover, 50 million people were recorded to be injured from RTAs in 2016 [11]. Currently more than 3000 people die from road traffic accidents and injuries worldwide every day [10].

Among the most affected group, motorcyclists take the lead due to the limited use of personal protective equipment compared to the other road users [12, 13]. Low and middle-income countries face more challenges as more than 85% of all deaths and 90% of all injuries of RTAs originated from this region [14]. Despite being the least motorized region, Africa has the highest road traffic fatality rates in the world: 26.6 deaths per 100,000 population [15]. With only 3% of world's registered vehicles, Africa alone contributes 20% of all RTAs globally. However, with 40% of world's registered vehicles, developed countries contribute only 7% of all RTAs globally [16].

Despite being characterized with the low level of safety, road infrastructure forms the dominant mode of transportation in Tanzania [17]. Currently there have been notable slowly decrease in RTAs trends in the country, in 2014, the recorded 15,420 RTAs caused 3,106 deaths and 15,230 injuries while in 2015 there were 8,777 RTAs with 3,574 deaths and 9,993 injuries. RTA is still a problem in the country as almost, 4000 people die annually from road crashes [18].

RTAs are influenced by many factors such as high speed, driving under influence of alcohol, and psychoactive substances, drivers’ fatigue, use mobile phone while driving, inadequate law enforcement, vehicle defect, and poor infrastructure (road) [19]. In Tanzania human factors account for 76.4% of all causes of road accidents [17].

Much has been done by the country to respond to road traffic accident. Currently, through road safety policy of 2009, Tanzania is in line with the United Nations Road Safety Collaboration (UNRSC) to implement the Decade of Action for road safety 2011–2020, a Global plan to integrate and coordinate global efforts to promote road safety. Noteworthy, under the UNRSC Tanzania is the first country to launch and implement the Ten Step Plan for safer road infrastructure, the program to reduce road casualty rate [20]. Improvement in traffic environment, provision of education and reliable information to the road users especially drivers are among the achieved efforts by the government. However, the country still experiences RTAs. Studies and reports showed motorcycles are the most contributor of RTAs as they accounted for 53.4% of all recorded accidents [12]. Dodoma is among the region in Tanzania that experiences the hardest hit from RTAs, in 2016 alone there were 310 RTAs which costed 144 lives and left 280 injured [18]. Since RTAs reflect the co-occurrence of multiple causes that involve road infrastructure, vehicle, and people, [21] and considering the information above, much have been done by the government to address the road infrastructure factor. This study therefore addresses the human factor gap by examining the level of compliance with road safety measures and the associated factors among commercial motorcyclists in Dodoma region.

Due to the ongoing road safety promotion efforts by the government, findings from this study provide the status of intermediate indicators that relate to RTAS and injuries prevention measures, which can be used for monitoring and evaluation research at region (Dodoma) and country level.

## 2 Methods

### 2.1 Study design and approach

The analytical cross-sectional study using quantitatively approach was conducted in Dodoma urban and Chamwino districts in Dodoma in 2020. Dodoma is among 31 administrative regions of Tanzania with population of 2,083,588 as per 2012 National Population and Housing Census.

### 2.2 Sampling and sample size estimation

This study targeted commercial motorcyclists located at registered parking stations in Dodoma urban and Chamwino districts, who were willing to participate and provided informed consent. A multistage sampling technique as employed by other studies [22, 23, 24] was used in this study. At first stage, purposive sampling was used to select two out of seven districts in Dodoma region. Dodoma urban and Chamwino districts were selected due to the ongoing increase in human population, a motor vehicle and particularly motorcycles. The increased was observed following an executive order by the Government of the United Republic of Tanzania to relocate its administrative operations from Dar es salaam to Dodoma in 2016.

In the second stage, simple random sampling, by lottery method was used to select six out of forty-one wards in Dodoma urban district. A total of sixty commercial motorcycle parking stations were assessed. In Chamwino district, three out of thirty-six wards were purposively selected due to their immediate vicinity to the highway. A total of fifteen commercial motorcycle parking stations were assessed. The employed sampling technique was cost and time-effective and ensured the study retains its randomness in sample selection at the second stage.

By using Fisher's formula below, a total of 382 motorcyclist were expected to be involved in the study, however, 4 participants failed to complete questionnaire. Therefore, a total of 378 participants were involved in the study.

$$N=\frac{\;{Z_{\propto /2}}^{2}\;\times P\times \left[1-P\right]}{{\text{ε}}^{2}}$$

Whereby:

N is the sample size.

$Z_{\propto /2}$ is the critical value of normal distribution at α/2 = 1.96.

P is the sample proportion = 53.4%, from the motorcycles proportion in road traffic accident [12].

εis the margin of error = 5%.

### 2.3 Data collection methods and techniques

The study used interviewer administered questionnaire with close -ended questions to collect information on the knowledge, attitude, practices, and compliance with road safety measures. The questionnaire was developed from three standardized road safety tools. It consisted of 60 questions itemized into five parts. The first part detailed the socio-demographic characteristics of study participants whereas second part focused on the level of compliance with road safety measures. Part three addressed questions that target knowledge level such as rightful age to drive and ride motor vehicle, legal requirement and necessity of driving school enrollment, and road sign adherence. Questions on the level of attitude (such as perception on tailgating and overtake from left side while riding) were addressed in part four. Part five covered questions regarding level of practice on adherence to road safety measures (such as helmet wearing, alcohol drinking and uses of mobile phone while riding) Knowledge measurements and questions were adapted from WHO tool of the second global status report on road safety of 2014. Socio-demographic, attitude and practice part were adapted from tool by Ranjan et al study in 2018 [25].

#### 2.3.1 Independent variables

##### 2.3.1.1 Socio-demographic information

The socio-demographic information collected included age, gender, marital status, level of education, residence, and working duration.

##### 2.3.1.2 Knowledge, attitude, and practice

The measurement scale of knowledge on compliance with road safety measures consisted of 13 items with the total score of 13. A score of 1 and 0 were given to the correct and wrong responses, respectively. The total score per respondent were converted to percent with the cut point of 50%. Inadequate knowledge was defined as having a score of 6 and below (50% and below 50%) and adequate knowledge was defined otherwise [11].

Likert-type scale used to measure the attitude of study participants. The scale had 12 items with 4 options (Strong disagree, disagree, agree, strongly agree) and a total of 48 score. The highest and lowest score per each question was 4 and 1, respectively. The computed mean of attitude after Likert scale analysis was 34. Commercial motorcyclists who scored less than 34 were considered to have negative attitude towards compliance with road safety measures, while those who scored above 34 were listed as participants with positive attitude toward the same.

Practice of commercial motorcyclist on adherence to road safety measures was measured by Likert-type scale with 10 items and 4 options per each item (Always, sometime, rarely, never). Per each item, the highest score was 4 and the lowest score was 1. The total score was 40 with the computed mean score of 30. Commercial motorcyclists who scored less than 30 were considered to have poor road safety practice, while those who scored above 30 were considered to have good road safety practice.

#### 2.3.2 Dependent variable

##### 2.3.2.1 Compliance with road safety measures

The level of compliance was measured by Likert-type scale consisting of 15 items with 4 options (Strong agree, agree, disagree, and strongly disagree) per each item and a total of 60 score. The highest and lowest scores for each item were 4 and 1 respectively, and the mean score was 35. Good compliance with road safety measures was defined as having a score of 35 and above and poor compliance was defined otherwise.

##### 2.3.2.2 Data processing and analysis

Data were analyzed using SPSS software, version 20. In the descriptive analysis, sociodemographic characteristics of study participants, and their level of knowledge, attitude, practice, and compliance were summarized in frequency and percentage and presented in tables.

Chi-square analysis test was used to assess the significance of difference in level of knowledge, attitude, practice, and compliance across sociodemographic characteristics of study participants. Since the study outcome is a binary variable, several approaches were used to examine whether socio-demographic characteristics, level of knowledge, attitude and practices were associated with compliance with road safety measures. First, bivariate analysis was performed to determine factors associated with the dependent variable. Subsequently, multivariable logistic regression was conducted to determine the predictors of good compliance with road safety measures. The model was applied with adjustment for socio-demographic information and other independent variables. The P-value and 95% confidence interval (CI) for the adjusted odds ratio (AOR) were used to confirm the significance of the association. In all analyses, a statistical significance was considered at p < 0.05 (2-tailed).

##### Statement of ethical approval

Ethical clearance for study and publication was sought from The University of Dodoma Institutional Research Review Committee.

## 3 Results

### 3.1 Socio-demographic characteristics of commercial motorcyclists

A total of 378 male commercial motorcyclists participated in this study; 34.7% from Chamwino district and 65.3% from Dodoma urban. Majority of participants 87.8% were aged between 18-29 years. Most of respondents (86.2%) lived in urban area. The education level of respondents ranged from primary, secondary to college, 51.9% of all respondents had secondary education as presented in Table 1.

**Table 1:** Socio-demographic characteristics of commercial motorcyclists in Dodoma region.

Variables	N = 378	
	n	%
**Residence**		
Chamwino	131	34.7
Dodoma Urban	247	65.3
**Age (Years)**		
18–29	332	87.8
30–49	45	11.9
50–69	1	0.3
**Gender**		
Male	378	100.0
Female	0	0.0
**Residence**		
Urban	326	86.2
Rural	52	13.8
**Marital status**		
Single	188	49.7
Married	190	50.3
**Education**		
Primary	84	22.2
Secondary	196	51.9
College and above	98	25.9
**Working Years' Experience**		
1–3	269	71.2
4–6	109	28.8
**Working days per week**		
1–2	0	0.0
3–4	0	0.0
5–7	378	100.0
**Working hours per day**		
6–10	8	2.1
11–14	149	39.4
15–19	170	45.0
20–24	51	

### 3.2 Level of knowledge, attitude, practice, and compliance with road safety measures among commercial motorcyclists

Out of 378 motorcyclists, 48.7% always wear helmet while on duty and 55.8% observed zebra mark for pedestrian users. Almost quarter (36.8%) of the motorcyclists were occasionally riding under alcohol influence, Table 2.

**Table 2:** Practice on compliance with road safety measures among commercial motorcyclists, Item analysis.

Item	N = 378			
	Always	Sometimes	Rarely	Never
	n (%)	n (%)	n (%)	n (%)
Wears helmet	184 (48.7)	52 (13.8)	113 (29.9)	29 (7.7)
Observes and obeys zebra mark	211 (55.8)	74 (19.6)	74 (19.6)	19 (5.0)
Carries ≥2 passengers at a time	61 (16.1)	112 (29.6)	148 (39.2)	57 (15.1)
Uses mobile phone while riding	39 (10.3)	156 (41.3)	102 (27.2)	81 (21.4)
Ensure passengers wear helmet	66 (17.5)	178 (47.1)	95 (25.1)	39 (10.3)
Drink alcohol and ride	19 (5.0)	139 (36.8)	64 (16.9)	156 (41.3)
Stops at a red traffic light	148 (39.2)	85 (22.4)	104 (27.5)	43 (11.3)
Wears reflective jackets	58 (15.1)	38 (10.1)	72 (19.0)	210 (55.6)
Observes roundabout rules	20 (5.3)	82 (21.7)	80 (21.2)	196 (51.9)
Wears open shoes while riding	70 (18.5)	186 (46.9)	89 (23.5)	33 (8.7)
Indicates before turning left/right	227 (61.0)	93 (23.6)	56 (14.8)	2 (0.5)

Table 3 presents results from item analysis which indicate the level measured knowledge, attitude, practice, and compliance with road safety measures among the interviewed study participants. Of the 378 commercial motorcyclists who were interviewed, only 61.9% had adequate knowledge on road safety measures and 59.5% had a positive attitude towards road safety measures. Nearly half of the participants (51.3%) were found to have good practice regarding road safety measures. However, it was alarming that only 39.7% of commercial motorcyclists were complying with road safety measures.

**Table 3:** Level of knowledge, attitude, practice, and compliance with road safety measures among commercial motorcyclists.

Variables	N = 378	
	n	%
**Knowledge**		
Inadequate knowledge	144	38.1
Adequate knowledge	234	61.9
**Attitude**		
Negative attitude	153	40.5
Positive attitude	225	59.5
**Practice**		
Poor road safety practice	184	48.7
Good road safety practice	194	51.3
**Compliance**		
Poor compliance	228	60.3
Good compliance	150	39.7

### 3.3 Association between level of compliance with road safety measures and knowledge, attitudes, practice, and socio demographic characteristics

Table 4 summarizes the associations between level of compliances with road safety measures and sociodemographic characteristics of study participants. There was no variation in compliance level based on residence of study participants. Married participants were more compliant with road safety measures than their counterpart (p < 0.001). Motorcyclists with over four years of experience reported to have more compliance with road safety measures than those with experience of one to three years (p < 0.05). However, there was no significant difference in the level of compliance with road safety measures across age group, level of knowledge and education and working hours per day.

**Table 4:** Association between level of compliance with road safety measures to knowledge, attitude, and practice levels of study participants.

Variables	Level of Compliance with road safety measures n = 378		P -Value
	Poor Compliance n (%)	Good Compliance n (%)	
**Residence**			
Rural	31 (59.6)	21 (40.4)	0.899
Urban	197 (60.4)	129 (39.6)	
**Age Group**			
18–29	203 (61.1)	129 (38.9)	0.422
30–69	25 (54.3)	21 (45.7)	
**Marital Status**			
Single	135 (71.8)	53 (28.2)	0.001
Married	93 (48.9)	97 (51.1)	
**Education Level**			
Primary	46 (54.8)	38 (45.2)	0.179
Secondary	127 (64.8)	69 (35.2)	
College and above	55 (56.1)	43 (43.9)	
**Working Hours Per Day**			
6–10 h	3 (37.5)	5 (62.5)	0.598
11–14 h	92 (61.7)	57 (38.3)	
15–19 h	102 (60)	68 (40)	
20–24 h	31 (60.8)	20 (39.2)	
**Working Years' Experience**			
1–3 years	172 (63.9)	97 (36.1)	0.028
4–6 years	56 (51.4)	53 (48.6)	
**Level of Knowledge**			
Inadequate	88 (61.1)	56 (38.9)	0.829
Adequate	140 (59.8)	94 (40.2)	
**Level of Attitude**			
Negative Attitude	104 (68.0)	49 (32.0)	0.014
Positive Attitude	124 (55.1)	101 (44.9)	
**Level of Practice**			
Poor road safety practice	146 (79.3)	38 (20.7)	0.001
Good road safety practice	82 (42.3)	112 (57.7)	

### 3.4 Multivariable logistic regression on the factors associated with good compliance with road safety measures

Table 5 details the comparison of compliance level to road safety measures across selected sociodemographic characteristics which suggested statistically significant variations by marital status and level of practice. Married motorcyclists were 2.86 times more likely to comply with road safety measures than single motorcyclists (p < 0.001). Participants with good practice regarding road safety measures had 5.13 times increasing odds of complying to road safety measures than those with poor road safety practice (p < 0.001). However, there were nonsignificant variation on the level of compliance by working years’ experience and attitude of motorcyclists. Motorcyclists with 4–6 years of working experience were 1.12 times more likely to comply with road safety measure than those who worked for only 1–3 years. Moreover, participants with positive attitude had 1.39 times increasing odds of complying with road safety measures than those with negative attitude.

**Table 5:** Multivariable logistic regression on the factors that associated with good compliance with road safety measures.

Variables	OR [95% CI]	P - Value	AOR [95% CI]	P - Value
**Marital Status**				
Single (Ref)	1.00		1.00	
Married	2.66 [1.73–4.07]	0.000	2.86 [1.75–4.67]	0.001
**Working Years' Experience**				
1–3 years (Ref)	1.00		1.00	
4–6 years	1.68 [1.10–2.63]	0.024	1.12 [0.69–1.94]	0.585
**Level of Attitude**				
Negative Attitude (Ref)	1.00		1.00	
Positive Attitude	1.73 [1.13–2.66]	0.012	1.39 [0.86–2.27]	0.182
**Level of Practice**				
Poor road safety Practice (Ref)	1.00		1.00	
Good road safety Practice	5.23 [3.32–8.29]	0.00	5.13 [3.16–8.32]	0.001

**OR** odds ratio; **CI,** confidence interval; **AOR,** adjusted odds ratio.

## 4 Discussion

The current study aimed to assess the level of compliance with road safety measures among commercial motorcyclists in Dodoma region. Though there is no culture taboo or law that prevent women from engaging to commercial motorcycling in Tanzania, all respondents were male, similar to what have been reported by other studies [23, 26, 27, 28, 29] that motorcycling is considered as male dominated occupation. This study showed that majority (87.8%) of the respondent were within the age range of 18–29 years, this is in consonance with findings obtained by [6, 14, 24] in Nigeria, Ghana and Ethiopia where youth constitute a great proportion of motorcyclists. Similarity may be accounted by the high level of unemployment among youth in developing countries forcing them to engage in commercial motorcyclist business.

In contrast to finding elsewhere [22, 30], results from this study revealed that only 39.5% of motorcyclist reported to have good compliance. However, compliance level differed within demographic characteristics of motorcyclists. Married group of commercial motorcyclists had 2.86 times increasing odds of complying with road safety measures than those who were not married. This finding can be explained by attitude, behavior, and practices of study participants towards road safety measures. Almost half (40.5% and 48.7%) of motorcyclists reported to have negative attitude towards road safety measures and poor road safety practice, respectively. Youth have been reported to comply with road safety measures mostly in the presence of traffic police officers or during police intervention [31, 32], and in this study 87.8% of study participants were youths aged between 18-29 years. Existing literature [5, 6, 14, 16, 24] elsewhere have reported youths, particularly men to be a more vulnerable group to the risk of road traffic accidents. Furthermore, enforcement helps to change attitudes towards poor road use, drivers are less likely to violate traffic laws when they know that they can be caught and fined [32]. Low level of compliance in our study might also be attributed to low enforcement of road safety measures to motorcyclist due to low number of police force deployed in rural road network and outskirt of the town. In 2016, from January to December there were a total of 2,210,739 traffic offenses reported in Tanzania and the routine administrative records reported a total of 4,721 traffic police officers in the police force. On average, one traffic police officer handled 468 traffic offences annually and in Dodoma region the ratio of traffic offences per traffic police officer was 312 in 2016 [18].

Mass campaigns such as “don't drink and drive” are important in enhancing community awareness towards road safety measures. Similarly to what have been reported by other studies elsewhere [30], in this study, a significant proportion of commercial motorcyclists (61.9%) had adequate knowledge on road safety measures. This finding can be attributed by the effort of government while implementing the decade of action for road safety (2011–2020) by providing education and reliable information to road users. Currently the Tanzania is on step to implement the ten steps approach of United Nations Road Safety Collaboration (UNRSC) [33].

Riding safely is considered as number one priority for a dedicated motorcyclists [34]. In this study more than half of respondents indicated to have a positive attitude towards road safety measures. Motorcyclist with positive attitude had 1.73 times increasing odds of complying with road safety measures than their counterpart group. Similar observation have been reported by other studies [35, 36] elsewhere. Desired intention towards a particular behavior or practices is determined by individual attitudes, being positive or negative regarding performing that behavior [37].

Compliance with road safety measure is determined by the individual good road safety practices [22, 38]. Similarly, to what has been reported elsewhere, results from this study indicated that almost half of respondents (48.7%) had poor road safety practices [24]. Moreover, motorcyclists with good road safety practices were 5.13 times more likely to comply with road safety measure than those with poor road safety practice. Helmet use was found among 48.7% of commercial motorcyclists in this study. Poor road safety practices increase the vulnerability of motorcyclists to injuries during accidents. A study on factors associated with road safety injuries in Tanzania revealed that motor-cycle crashes accounted for great number of victims with head injuries (54.3%), fractures (52.9%) and multiple injuries (51.2%) and furthermore, majority (80.2%) of those with head injuries had no helmet [12]. This finding underlines the need to enforce road safety regulations to all road users with special focus on motorcyclists.

## 5 Conclusion

Finding from this study revealed that human factors including, positive attitude and good practice on road safety measures, many years of experience in riding motorcyclists and marital status (being married) were associated to compliance with road safety measure among commercial motorcyclist in Dodoma region. Coordinated approaches are needed to improve the way motorcyclist ride and behave on road. This must include among others, continuing provision of road safety education and enforcement of road safety measures to change attitudes towards poor road use and prevent unlawfully, dangerous, and irresponsible behaviors among commercial motorcyclists. Despite road safety being everyone's responsibility, experienced commercial motorcyclists with clean riding records can be used as the champions to influence others. The target must be the novice in motorcycling business and those with few years of working experience. Moreover, the study recommend improvement in the quality, management, and accessibility of road safety data by the responsible ministry, as the basis for evidence-based interventions.

### 5.1 Limitations of the study

Motor vehicle characteristics including road-worthiness system (lights, brakes), vehicle types (brand names), engine capacity, and maximum load capacity are basic component of road safety elements, our data collection tool did incorporate this component, thus, we were unable to determine the contribution of vehicle factor in our study.

Commercial motorcyclists were the targeted study population, regardless of their sex category. We did not find women motorcyclist within the study area; thus, we were unable to stratify analysis by sex category. It may be beneficial for future study to purposeful involve women to provide greater insights into road safety measures compliance based on the sex of study participants.

Finally, the data collected from commercial motorcyclists were based on self-reports that are likely to be subject to social desirability. Commercial motorcycling is considered a worthy business, generates income for the operators and owners, as a result, there is a limit to which such responses can be considered accurate, since study participants want to be accepted and seeking social approval hence might have failed to communicate how they truly feel about questions being asked.

## Declarations

### Author contribution statement

Jackson Silvano Nzuchi, Selestin Joseph Ngoma: Conceived and designed the experiments; Performed the experiments; Analyzed and interpreted the data; Wrote the paper.

Eugene Benjamin Meshi: Conceived and designed the experiments; Analyzed and interpreted the data; Wrote the paper.

### Funding statement

This research did not receive any specific grant from funding agencies in the public, commercial, or not-for-profit sectors.

### Data availability statement

Data will be made available on request.

### Declaration of interest's statement

The authors declare no conflict of interest.

### Additional information

No additional information is available for this paper.

## Appendix A Supplementary data

Questionnaire.pdf Questionnaire.pdf
