# Supplementary material for: Commercial motorcyclists and road safety measures compliance. A case study of Dodoma city, central Tanzania
Source: Heliyon. 2022 Aug 19;8(8):e10297. doi: 10.1016/j.heliyon.2022.e10297 (PMC9424942; doi:10.1016/j.heliyon.2022.e10297)
Supplement: Questionnaire.pdf [file mmc1.pdf]

Questionnaire No:……………   Date: ………………….

KNOWLEDGE, ATTITUDE AND PRACTICE ON COMPLIANCE WITH ROAD SAFETY MEASURES AMONG COMMERCIAL MOTORCYCLISTS IN DODOMA REGION OF TANZANIA

English version

PART I; Socio-Demographic Characteristics of the Respondents

1) Respondent's address; tick (✓) at the appropriate

   □ Dodoma City

   □ Chamwino district

2) What is your age? Write in the bracket at the right side (…………)

Tick (✓) in the box where is appropriate to you

3) What is your sex?

   □ Male

   □ Female

4) Where are you living? (Home or resident)

   □ Rural

   □ Urban

5) What is your marital status?

   □ Single

   □ Married

6) What is your level of education?

   □ No formal education

   □ Primary

   □ Secondary

   □ College and above

7) How long have you been engaged in commercial motorcycling business? Write number of years of experience in the brackets (……….)

8) How many days do you ride motorcycle per week? Answer in the brackets (……….)

9) How many hours do you spend in riding commercial motorcycle per day? Answer in the brackets (…………)

PART II: Measuring compliance with road safety measures among commercial motorcyclists

In the table below you are provided with several statements, please tick (✓) in the box where you think is applicable to you

Statements	Strong agree	Agree	Disagree	Strong disagree
10) You attended accredited driving school for trainings				
11)You own a valid driving license				
12) You ride a motorcycle without helmet				
13) You carry a passenger who do not wear helmet on your motorcycle				
14) You don't observe road signs while riding a motorcycle				
15). Uses mobile phone while riding				
16). You do not own a motorcycle helmet				
17). You do not wear reflective jackets while riding				
18). You do carry more than one passenger on motorcycle at a time				
19). Sometimes you ride a motorcycle above speed limit				
20). You do not indicate before turning left/right				
21). You ride a motorcycle even after taking alcohol				
22). You do not wear safety boots while riding a motorcycle				
23). You always overtake from right side while on motorcycle				
24). You do not wear safety goggles when riding a motorcycle				

PART III; Measuring knowledge on compliance with road safety measures among commercial motorcyclists

Please tick (✓) on "YES" section if you agree with the statement, and on "NO" if you disagree with the statement

Statements	Yes	No
25). In Tanzania, for a driver to own a driving license must be at the age of 18 years old and above		
26). It is compulsory to join a driving school before been awarded a valid driving license.		
27) Motorcycle helmets are personal protective gears to the motorcyclists		
28). Motorcyclist must indicate when making a turn.		
29). It is illegal to ride a motorcycle without wearing helmet		
30). Wearing reflective jacket to motorcyclist increase the visibility to other road users		
31). It is important for motorcyclist to wear an eyeglass as a protective gear		
32). To ride a motorcycle in high speed increases the chance for road accident occurrence		
33). It is strictly prohibited using mobile phone while riding		
34). A motorcyclist, must always wear safety boots while riding		
35). A motorcyclist must give a passenger helmet before riding.		
36). Zebra crossing sign indicate a prime priority must be given to pedestrians first		
37). Concerning alcohol blew test for drivers and riders, the purpose is to reduce occurrence of road traffic accidents		
38). Red traffic light requires all vehicles to stop and wait, they can only proceed when the traffic light turns green		

PART IV: Measuring Attitude on compliance with road safety measures among commercial motorcyclists

You have provided a list of statements in the table below, please tick (✓) in the box where you think is appropriate to you.

Statements	Strong disagree	Disagree	Agree	Strong agree
39) Drinking while riding increases the risk of road accidents occurrences				
40). Tailgating can easily cause collision between two vehicles				
41). Itis prohibited to overtake from left side while riding a motorcycle				
42). High speed is one of main cause of road accidents among motorcyclists				
43). It is necessary for a motorcyclist to own a driving license				
44). Uses of mobile phone while riding contributes to the occurrence of road accidents				
45). Road signs and symbols help to reduce road traffic accidents				
46). Pedestrians should be given a prime priority at zebra mark to cross				
47). Zebra crossing are rarely observed by motorcyclist				
48). Helmet becomes a protective gear when properly worn by the motorcyclist while riding				
49). Complying to traffic lights is the wastage of time to commercial motorcyclists				
50). Motorcyclist must always wear reflective jackets for easy visibility from other road users				

PART V: **Measuring Practices on compliance to road safety measures among commercial motorcyclists**

In the table below, you are provided with various statements, please tick (✓) in the box where you think is appropriate to you:

How often do you do the following?

Statements	Always	Sometimes	Rarely	Never
51). Wearing helmet while riding				
52). Observes and obeys zebra mark for pedestrians to cross				
53). Carry more than one passenger at a time				
54). Uses mobile phone while riding				
55). Ensure passengers wear helmet				
56). Drink alcohol and ride?				
57). Stop at red traffic light?				
58) Wearing reflective jacket while riding?				
59). Observes roundabout rules?				
60). Indicates before turning left/right?				

THANK YOU FOR YOUR COOPERATION.
